# Intramolecular hydroxycarbene C–H-insertion: The curious case of (*o*-methoxyphenyl)hydroxycarbene

**DOI:** 10.3762/bjoc.6.121

**Published:** 2010-11-11

**Authors:** Dennis Gerbig, David Ley, Hans Peter Reisenauer, Peter R Schreiner

**Affiliations:** 1 Institut für Organische Chemie, Justus-Liebig University Giessen, Heinrich-Buff-Ring 58, 35392 Giessen, Germany

**Keywords:** benzofuran, C–H-insertion, hydroxycarbene, singlet carbene, tunneling

## Abstract

The first C–H insertion of a hydroxycarbene species in the gas phase has been observed experimentally by means of high vacuum flash pyrolysis (HVFP) and subsequent matrix isolation: (*o*-Methoxyphenyl)glyoxylic acid gives non-isolable (*o*-methoxyphenyl)hydroxycarbene upon pyrolysis at 600 °C, which rapidly inserts into the methyl C–H bond. The insertion product, 2,3-dihydrobenzofuran-3-ol, was trapped in an excess of Ar at 11 K and characterized by infrared spectroscopy. The insertion process kinetically outruns the alternative [1,2]H-tunneling reaction to *o*-anisaldehyde, a type of reaction observed for other hydroxycarbenes. Traces of the dehydration product, benzo[*b*]furan, were also detected. The potential energy hypersurface including the insertion and hydrogen migration processes was computed at the all-electron coupled-cluster level of theory encompassing single and double substitutions and perturbatively included triple excitations [AE-CCSD(T)] in conjunction with a correlation-consistent double-ζ basis set (cc-pVDZ) by utilizing density functional theory (DFT) optimized geometries (M06-2X/cc-pVDZ) with zero-point vibrational energy (ZPVE) corrections. Exchange of the methoxy for a trifluoromethoxy group successfully prevents insertion and (*o*-trifluoromethoxy)benzaldehyde is produced instead; however, the carbene cannot be observed under these conditions. Thermal decomposition of (*o*-methoxyphenyl)glyoxylic acid in refluxing xylenes does not give the insertion product but yields *o*-anisaldehyde. This unanticipated outcome can be rationalized by protonation of the hydroxycarbene intermediate leading to the tautomeric formyl group. Thermochemical computations at M06-2X/cc-pVDZ in conjunction with a self-consistent solvent reaction field model support this suggested reaction pathway.

## Introduction

**Scheme 1:**
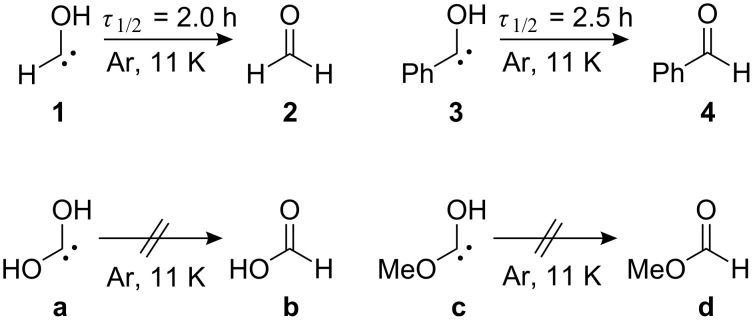
Unimolecular reactivity of hydroxycarbenes under cryogenic conditions: [1,2]H-Tunneling of **1** and **3** (*τ*_1/2_: half-life).

Hydroxycarbenes have been the subject of many theoretical and experimental studies since the early years of last century, however, these proved to be elusive for a long time [1]. Whilst a large variety of hydroxycarbene ligands have been prepared as fairly stable Fischer-type carbene–metal complexes with group VI, VII, VIII, and X elements [2–12] it was only very recently that free hydroxycarbenes were generated by high vacuum flash pyrolysis (HVFP) followed by immediate matrix isolation and thoroughly characterized by means of IR- and UV-spectroscopy [13–15]. Very surprisingly, several hydroxycarbenes exhibit remarkable [1,2]H-tunneling under cryogenic conditions in solid noble gas matrices, even at temperatures as low as 11 K: Hydroxymethylene (**1**) [13] and phenylhydroxycarbene (**3**) [14] yield formaldehyde (**2**) and benzaldehyde (**4**), respectively, as a result of facile [1,2]hydrogen tunneling from the hydroxy group to the carbene center. Dihydroxycarbene (**a**) [15] and methoxyhydroxycarbene (**c**) [15], however, do not undergo [1,2]H-tunneling under the same conditions: Their respective products, formic acid (**b**) and methyl formate (**d**) were not detected in matrix isolation experiments (Scheme 1).

**Scheme 2:**
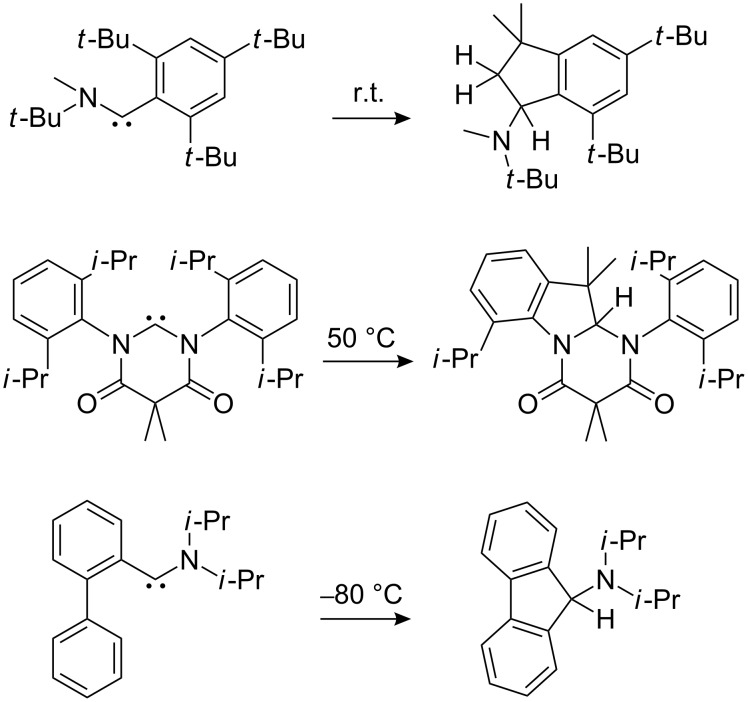
A selection of heterocarbenes that undergo intramolecular C–H insertions.

**Scheme 3:**
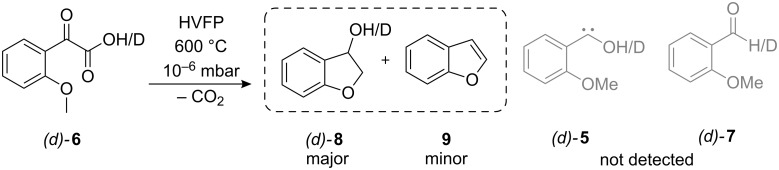
Attempted generation of **5** and *d*-**5** as well as their corresponding insertion products.

Ring insertions characteristic for other singlet phenylcarbenes, i. e., phenylmethylcarbene [16] and phenylchlorocarbene [17], were not experimentally observed for **3**, and we report herein the first C–H-bond insertion reaction of a hydroxycarbene that is akin to other heterocarbenes [18–20] (Scheme 2). (*o*-Methoxyphenyl)hydroxycarbene (**5**) serves as the model compound for studying the intramolecular carbene C–H-bond insertion both under matrix isolation and solution conditions (Scheme 3). The generation of such carbenes in solution in high-boiling solvents would also provide convenient preparative access to dihydrobenzofuranols from readily accessible α-keto acids as the starting materials. Our results were rationalized by quantum chemical computations.

## Results and Discussion

### Matrix isolation studies on (*o*-methoxyphenyl)hydroxycarbene

In the course of our ongoing investigations regarding the nature of the fascinating [1,2]H-tunneling mechanism in phenylhydroxycarbenes (with parent **3**, Scheme 1), we sought to study the behavior of derivatives of **3** in Ar matrices at temperatures as low as 11 K. We attempted to generate novel *o*-methoxy-substituted carbene **5** by extrusion of carbon dioxide from (*o*-methoxyphenyl)glyoxylic acid (**6**) by HVFP and subsequent condensation and isolation of the pyrolysis products in an excess of Ar (Scheme 3). However, neither **5** nor its tunneling product, *o*-anisaldehyde (**7**), could be detected: Comparison with an authentic spectrum of matrix-isolated **7** showed that this compound was not present among the pyrolysis products. Even after more than 12 h in the dark at 11 K, the IR spectrum was unchanged, indicating that there is no compound among the pyrolysis products that is susceptible to a tunneling decay mechanism. The TD-DFT (B3LYP/cc-pVDZ) absorption maximum of **5** lies at 591 nm [21–22]. To rule out the existence of a persistent, non-tunneling carbene, irradiation experiments were conducted: Subsequent irradiation with a high-pressure Hg-lamp at λ = 577 nm, 546 nm, and 313 nm did not lead either to the appearance of new or the disappearance of existing signals, thereby verifying the complete absence of **5**.

**Scheme 4:**
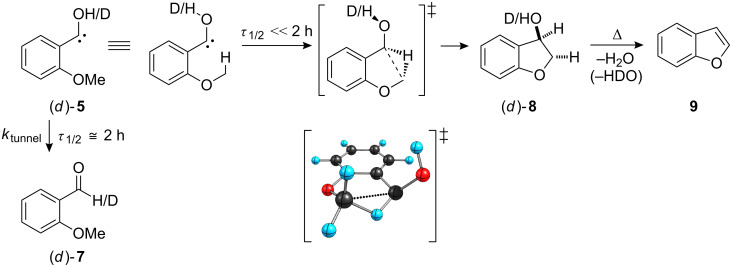
Proposed mechanism for the generation of **8** and **9**. The [1,2]H-tunneling process apparently cannot compete with C–H-insertion (*τ*_1/2_: half-life).

Analysis of the IR spectrum showed that **8** was the main product instead. To confirm the C–H-insertion into the neighboring methyl group, to yield 2,3-dihydrobenzofuran-3-ol (**8**), a sample of **8** was prepared by the reduction of commercially available 3-coumaranone and subjected to matrix isolation studies. All intense signals in the original pyrolysis spectrum of **6** could be shown to originate from **8**. As a side product, traces of the dehydration product of **8**, benzo[*b*]furan (**9**), were found and identified by comparison with an authentic spectrum of matrix-isolated **9** (Scheme 3 and Scheme 4).

**Figure 1:**
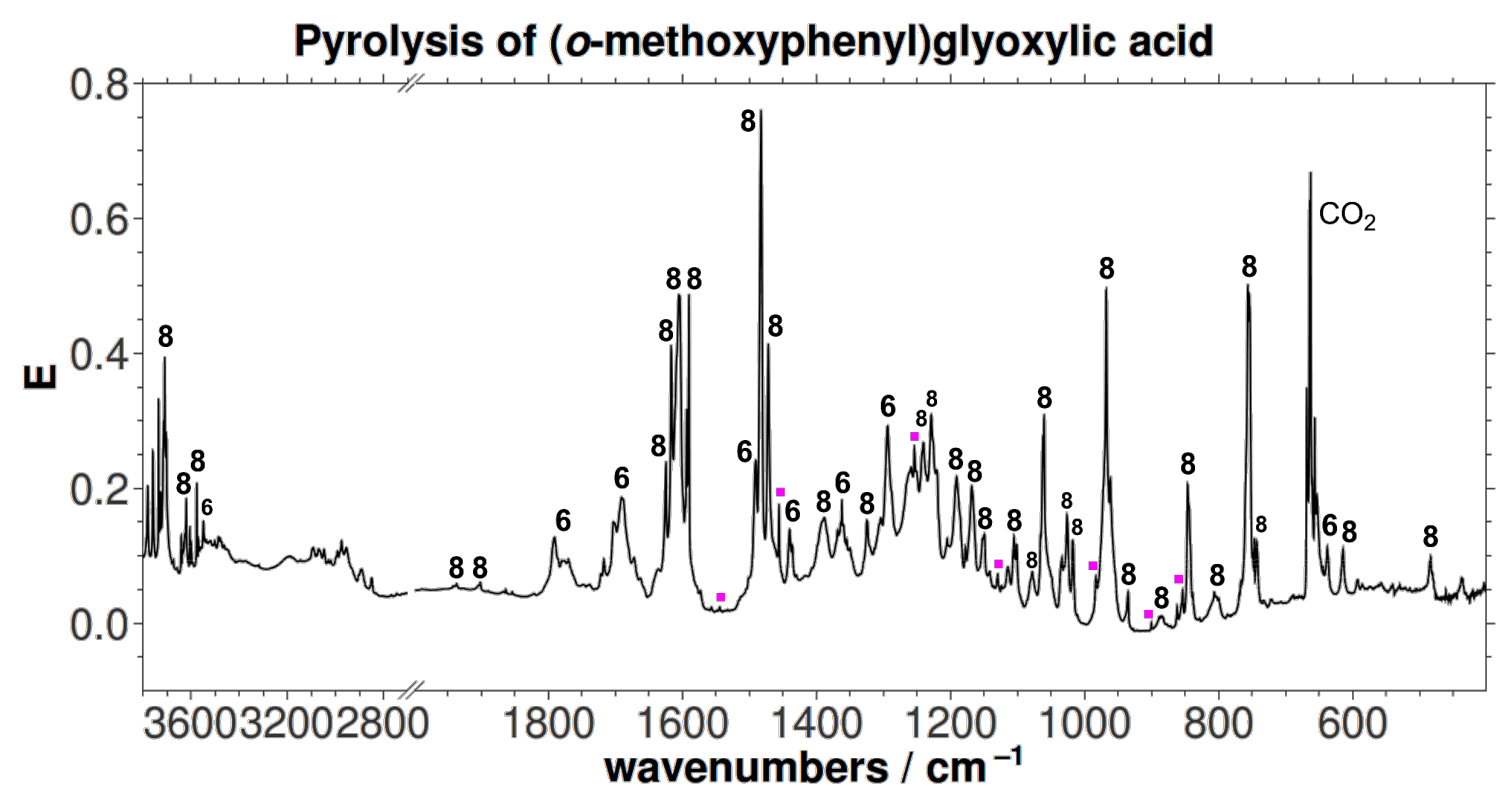
Unmodified matrix IR spectrum (Ar, 11 K) of the pyrolysis (600 °C) of **5**. Traces of **9** are indicated by magenta dots.

**Figure 2:**
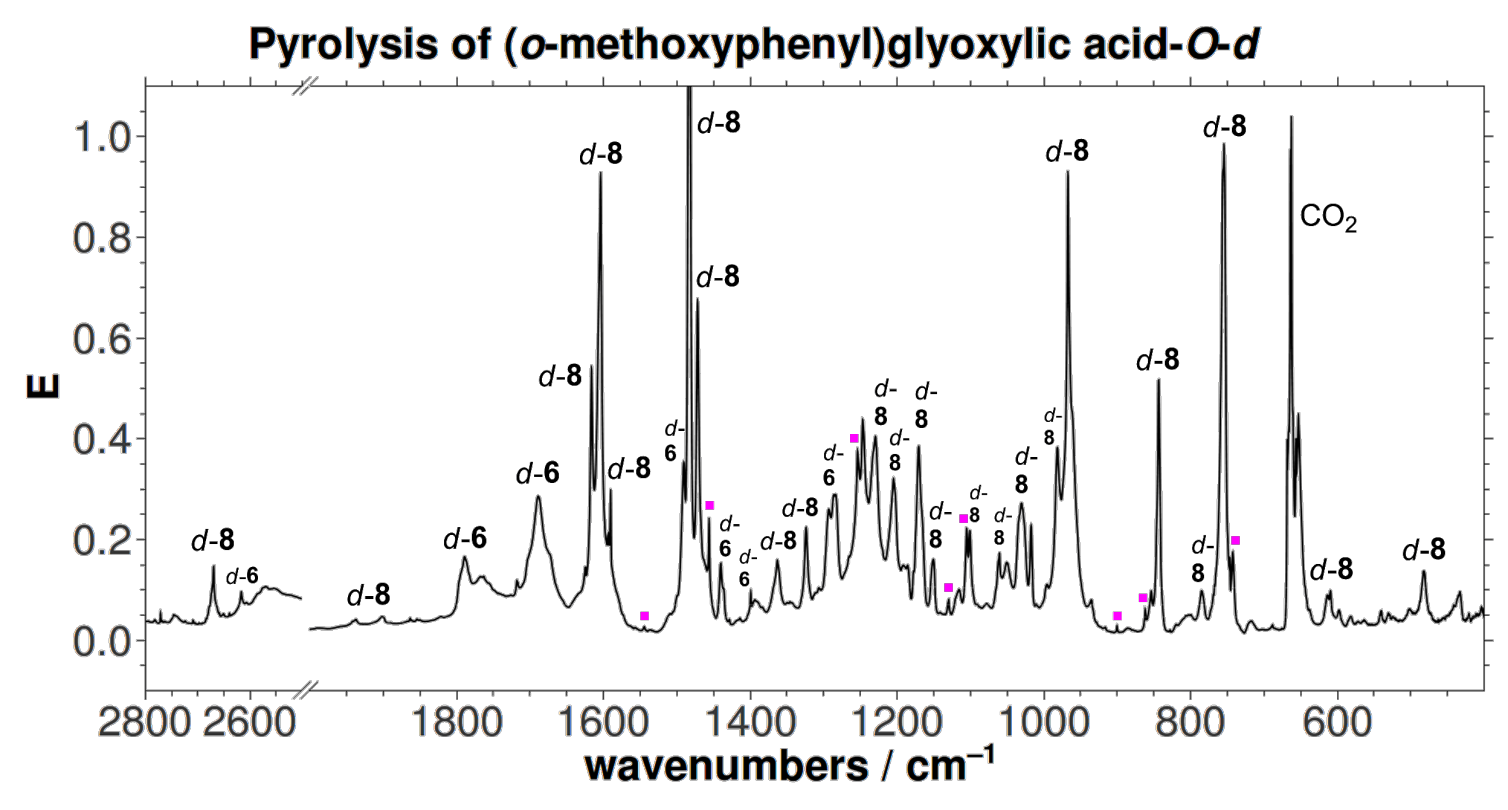
Unmodified matrix IR spectrum (Ar, 11 K) of the pyrolysis (600 °C) of *d*-**5**. Traces of **9** are indicated by magenta dots.

The [1,2]H-tunneling reactions in hydroxycarbenes can be suppressed by an exchange of hydrogen for deuterium. Hence, carbene signals can readily be identified by prolonged irradiation at or near the maximum absorption wavelength of the carbene. Thus, the pyrolysis experiment was repeated with the mono-deuterated acid *d*-**6** (*o*-MeOC_6_H_4_COCOOD) to yield *d*-**8** (OD). Again minor amounts of **9** (without any deuterium incorporation) were formed. In accordance with prior results, neither *d*-**5** nor *d*-**7** could be detected after irradiation. A proposed mechanism for the formation of the insertion product **8** is presented in Scheme 4. Matrix isolation spectra of both pyrolyses (**6** and *d*-**6**, respectively) are presented in Figure 1 and Figure 2, along with an assignment of all signals. A collection of all the matrix spectra is contained in Supporting Information File 1.

**Scheme 5:**
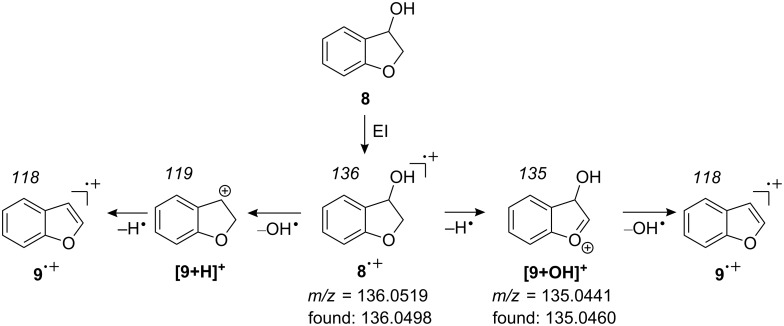
Decay of the 2,3-dihydrobenzofuran-3-ol molecular radical cation (**8^+•^**).

After warming of the matrix to room temperature, a sample of the pyrolysis products of **6** was collected from the matrix window. The molecular ions of **8** and **9** were found in the corresponding electron-impact mass spectrum, most probably generated through the decay mechanism proposed by Florêncio et al. [23] (Scheme 5).

### Matrix isolation studies on [(*o*-trifluoromethoxy)phenyl]hydroxycarbene

**Scheme 6:**
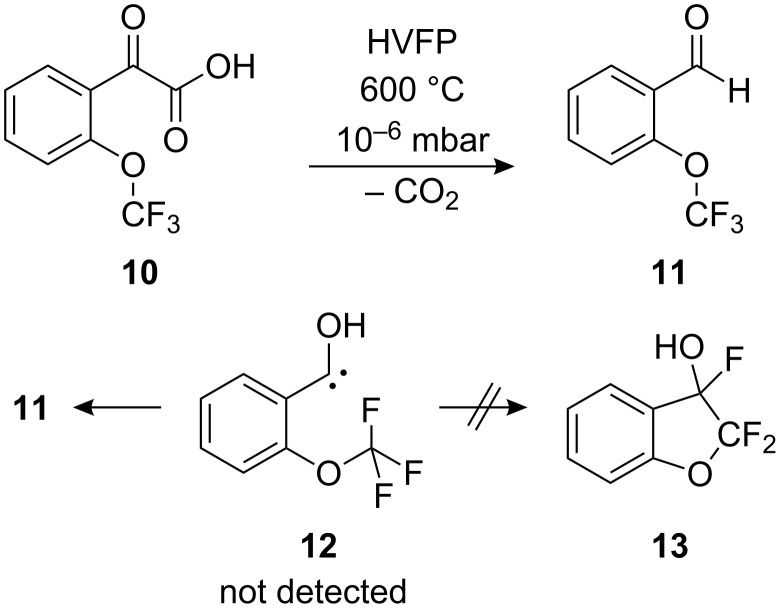
Attempted generation of **12** and the actual pyrolysis product **11**.

Exchanging the methoxy group for a trifluoromethoxy moiety should prohibit the insertion reaction due to the much stronger carbon–fluorine bond compared to the carbon–hydrogen bond. HVFP of [(*o*-trifluoromethoxy)phenyl]glyoxylic acid (**10**) at 600 °C (500 °C, 800 °C) and subsequent matrix isolation gave (*o*-trifluoromethoxy)benzaldehyde (**11**); as expected, no insertion product (**13**) was detected (Scheme 6).

Contrary to our findings on hydroxycarbenes, [(*o*-trifluoromethoxy)phenyl]hydroxycarbene (**12**) was not detected. Instead, only the substituted anisaldehyde **11** could be identified. On repeating the experiment with deuterated acid (OD) *d*-**10**, no deutero-carbene *d*-**12** was likewise produced, as verified by subsequent irradiation (577 nm, 313 nm). The computed [1,2]H-tunneling half-lives of **5** and **12**, based on a) an Eckart barrier approach, and b) the Wentzel–Kramers–Brillouin (WKB) approximation (see Computational methods for details), are summarized in Table 1, together with the computed half-life of **3** for comparison.

**Table 1:** Computed half-lives (*unscaled*) for the [1,2]H-tunneling reaction in carbenes **3**, **5**, and **12** at 11 K. The measured half-life of **3** is 2.5 h at 11 K. The thermal barrier for the [1,2]H-shift was computed at AE-CCSD(T)/cc-pVDZ // M06-2X/cc-pVDZ; (cf. Computational methods).

	∆*H*^‡^ for [1,2]H-shift [kcal mol^–1^]	Eckart [h]	WKB [h]

PhCOH (**3**)	28.9	3.7	4.8
*o*-MeOC_6_H_4_COH (**5**)	27.1	0.6	0.5
*o*-F_3_COC_6_H_4_COH (**12**)	29.3	1.2	1.1

Based on the computed tunneling half-lives, **12** should be observable in matrix-isolation experiments if it survives the formation conditions in the gas phase. The same holds true for *d*-**12**.

### Pyrolysis of (*o*-methoxyphenyl)glyoxylic acid (6) in solution

**Scheme 7:**
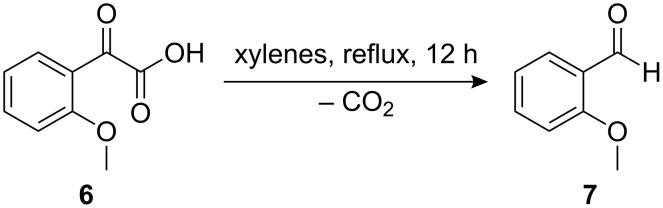
Unanticipated reaction of **6** upon heating in xylenes.

Surprisingly, the reactivity of **5** in solution is quite different from that in our matrix isolation experiments as *o*-anisaldehyde **7** forms instead of **8** upon refluxing **6** in xylenes (bp 139–140 °C) for 12 h in almost quantitative yield (Scheme 7). This unanticipated outcome is in stark contrast to the reactions of other heterocarbenes that have been shown to insert into comparable methyl C–H bonds in solution with ease [24]. When the reaction was carried out in an even higher boiling solvent, i.e., nitrobenzene (bp 211 °C), a complicated mixture of decomposition products resulted.

In order to probe for a chemical connection between **7** and **8**, insertion product **8** was also refluxed in xylenes for 12 h. However, only pure **8** was recovered. As expected on thermodynamic and kinetics grounds, the direct interconversion of **7** and **8** can therefore be excluded.

### Computational considerations

**Scheme 8:**
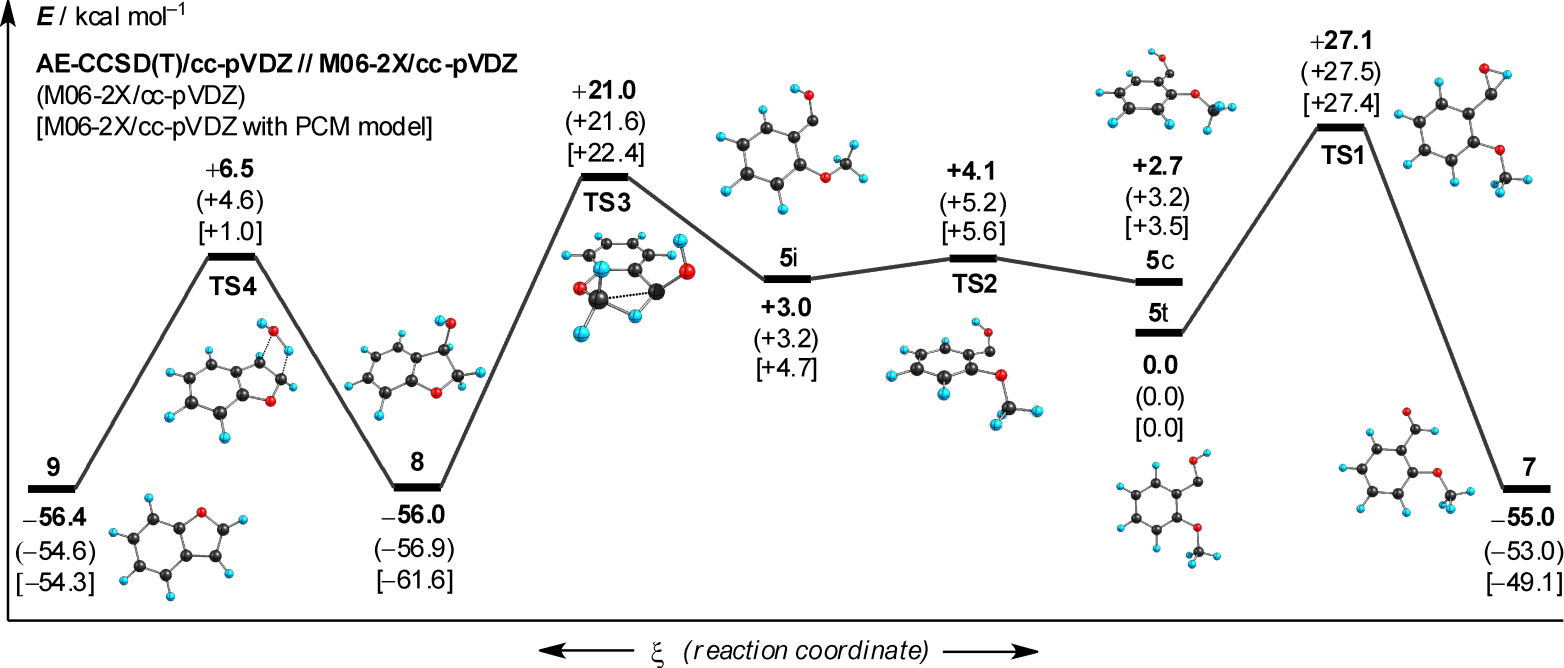
Potential energy hypersurface of (*o*-methoxyphenyl)hydroxycarbene (**5**) (not drawn to scale; ZPVE included); *legend*: **5c**: carbene, *cis* conformation; **5t**: carbene, *trans* conformation; **5i**: conformer that undergoes insertion.

AE-CCSD(T)/cc-pVDZ single point computations (with DFT ZPVEs; Scheme 8, for computational details see Computational Methods below) on M06-2X/cc-pVDZ geometries do not indicate a thermodynamic preference for either **7** or **8**. The thermal barrier for the reaction of **5** via conformer **5i** leading to **8** is lower (18 kcal mol^–1^, via **TS3**) than that leading to **7** (27 kcal mol^–1^, via **TS1**). These results help rationalize the observed reactivity under HVFP conditions. The thermal barrier for the dehydration of **7** is quite high (in excess of 60 kcal mol^–1^, via **TS4**), which was confirmed by HVFP of **7** at 600 °C and subsequent matrix isolation of the products: While **8** was more abundant at this temperature than in the pyrolysis of **6**, **7** was for the most part unchanged. In order to assess the condensed phase reactivity of **5**, M06-2X/cc-pVDZ computations including a solvent model were performed. These indicate a thermodynamic preference of −12 kcal mol^–1^ for the insertion product **8** over the aldehyde **7** as well as a 5 kcal mol^–1^ lower activation energy for the formation of **8**.

**Scheme 9:**
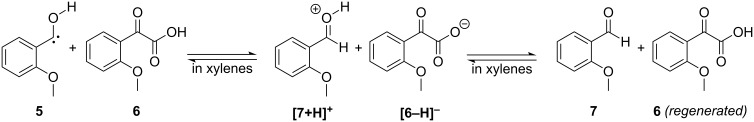
Acid-catalyzed generation of **7** by unreacted **6**.

Based on this potential energy hypersurface, it seems surprising that **7** is produced by pyrolysis of **6** in the condensed phase as **8** is both favored both thermodynamically and kinetically due to a lower thermal barrier. As a result, another mechanism must be implicated for the transformation of **6** to **7**. A possibly alternative pathway, involving an acid-catalyzed formation of the aldehyde, is presented in Scheme 9: Protonation of **5** at the carbene center by **6** leads to a carbonyl-protonated aldehyde **[7+H]^+^**, which is then deprotonated at the oxygen atom in the follow-up reaction, regenerating **6** and producing **7**. As the proton affinity *E*_pa_ of **5** was computed to be 262 kcal mol^–1^, which is comparable to those of alkali metal hydroxides, this seems a viable possibility.

## Conclusion

The formation of a 2,3-dihydrobenzofuran derivative from carbene **5** in the gas phase is the first C–H insertion reaction observed for a hydroxycarbene derivative. Hence, the general reactivity pattern of **5** under HVFP conditions is similar to that of other hetereocarbenes. However, synthetic access to substituted 2,3-dihydrobenzofuran derivatives through the in situ generation of various (*o*-methoxyphenyl)hydroxycarbenes from (*o*-methoxyphenyl)glyoxylic acids in high-boiling solvents is not possible, because an *o*-anisaldehyde derivatives are formed. We suggest that this dichotomy in reactivity between the high-temperature HVFP and the high-temperature solution experiments derives from protonation of the intermediate hydroxycarbene by the starting material in solution; this inevitably leads to the aldehyde upon deprotonation. Although preliminary tunneling computations indicate that carbene **12** should be observable, it has not yet been detected experimentally.

## Experimental

**Matrix-isolation studies.** An APD Cryogenics HC-2 closed-cycle cryostat system with an inner CsI window was used for IR measurements. Spectra were recorded with a Bruker IFS 55 FT-IR spectrometer (4500–300 cm^–1^ spectral range with a resolution of 0.7 cm^–1^). For the combination of high-vacuum flash pyrolysis with matrix isolation, a small, custom-built, water-cooled oven was used, which was directly connected to the vacuum shroud of the cryostat. The pyrolysis zone consisted of an empty quartz tube with an internal diameter of 8 mm and a length of the heating zone of 50 mm, which was resistively heated by a coax wire. The temperature was controlled with a Ni/CrNi thermocouple. (*o*-Methoxyphenyl)glyoxylic acid (**6**) was evaporated at room temperature from a small storage tube into the pyrolysis tube. All pyrolysis products were immediately co-condensed with a large excess of argon (typically 30 to 80 mbar from a 2000 mL storage bulb) on the surface of the 11 K matrix window at a distance of approximately 50 mm. A high-pressure mercury lamp (HBO 200, Osram) with a monochromator (Bausch & Lomb) was used for irradiation. Experiments with deuterated acid were conducted accordingly.

**(*o*-Methoxyphenyl)glyoxylic acid (6)** [25]. To a solution of 3.00 g (20.0 mmol) *o*-methoxyacetophenone in absolute pyridine, was added 3.33 g (30.0 mmol) of selenium dioxide. The mixture was stirred at 80 °C for 4 h. After filtration, concentration of the solution gave a brown oil that was dissolved in 5% sodium hydroxide solution and washed three times with small portions of diethyl ether. The aqueous layer was acidified with dilute hydrochloric acid and extracted with ethyl acetate. The organic layer was then dried over sodium sulfate. Filtration and concentration gave a brown oil, which crystallized on standing. (*o*-Methoxyphenyl)glyoxylic acid (3.30 g,18.4 mmol) was obtained as a brown solid. Sublimation in vacuo afforded the pure title compound as a white to yellowish powder in 60% yield.

^1^H NMR (400 MHz, *d*_6_-DMSO): δ = 3.85 (s, 3H, –OMe), 7.13 (t, 1H, ^3^*J* = 7.5 Hz, *p*-H to –OMe), 7.24 (d, 1H, ^3^*J* = 8.4 Hz, *o*-H to –OMe), 7.66–7.78 (m, 2H), 14.01 (s, 1H, acid-H); ^13^C NMR (100 MHz, *d*_6_-DMSO): δ = 56.2 (–OMe), 113.1 (*o*-C to –OMe), 121.2 (*p*-C to –OMe), 122.2 (*ipso*-C to –COCOOH), 126.1 (*o*-C to –COCOOH), 136.6 (*p*-C to –COCOOH), 160.0 (*ipso*-C to –OMe), 166.8 (–CO*C*OOH), 188.1 (–*C*OCOOH).

**(*o*-Methoxyphenyl)glyoxylic acid-*O*-*d*** (*d*-**6**) was obtained by repeated dissolution of (*o*-methoxyphenyl)glyoxylic acid in excess deuterium oxide followed by evaporation of the solvent in vacuo.

**2,3-Dihydrobenzofuran-3-ol (8)** [26]. To a solution of 1.96 g (5 mmol) 3-coumaranone in 40 mL of absolute methanol, sodium borohydride was added in small portions at –10 °C until the solution solidified. After the succession of hydrogen evolution, 20 mL of 0.2 N hydrochloric acid was added to the reaction mixture. The mixture was then extracted with chloroform. The combined organic layers were washed with brine and dried over anhydrous sodium carbonate. Filtration and removal of the solvent gave a brown oily liquid that was immediately purified by flash chromatography on a short column of silica gel with *tert*-butyl methyl ether as the eluent. The purified product was obtained as the second fraction (*R*_f_ ≈ 0.6) in 30% yield.

^1^H NMR (400 MHz, CDCl_3_): δ = 1.91 (s, 1H, –OH), 4.35 (dd, 1H, ^2^*J* = 10.6 Hz, ^3^*J* = 2.5 Hz, –O–C*H*_2_–), 4.44 (dd, 1H, ^2^*J* = 10.6 Hz, ^3^*J* = 6.5 Hz, –O–C*H*_2_–), 5.26 (dd, 1H, ^3^*J* = 6.5 Hz, 2.5 Hz,–C*H*OH–), 6.81 (d, 1H, ^3^*J* = 8.1, *o*-H to –O–CH_2_–), 6.87 (t, 1H, ^3^*J* = 7.04, *p*-H to –O–CH_2_–), 7.17-7.23 (m, 1H, *p*-H to –CHOH–), 7.34 (d, 1H, ^3^*J* = 7.5 Hz, *o*-H to –CHOH–); ^13^C NMR (100 MHz, CDCl_3_): δ = 106.5 (–O–CH_2_–), 111.4 (–CHOH–), 121.4 (*o*-C to –O–CH_2_–, *m*-C to –CHOH–), 122.7 (*p*-C to –O–CH_2_),124.2 (*o*-C to –CHOH–, *m*-C to –O–CH_2_–), 127.4 (*ipso*-C –CHOH–), 144.9 (*p*-C to –CHOH–), 154.9 (*ipso*-C –O–CH_2_–).

**2,3-Dihydrobenzofuran-3-ol-*O*-*d*.** H–D-exchange with D_2_O proved to be unsuccessful on the preparative scale. As a consequence, the deuterated compound was prepared in the same way as its protium analogue: To a solution of 0.55 g (1.3 mmol) of 3-coumaranone in 20 mL of methanol-*O*-*d*, sodium borohydride was added in small portions at −10 °C until the solution solidified. After the succession of hydrogen evolution, 10 mL of D_2_O were added to the reaction mixture. The mixture was then extracted with CDCl_3_. The combined organic layers were washed with brine and dried over anhydrous sodium carbonate. Filtration and removal of the solvent gave a yellow liquid in 60% yield.

^1^H NMR (400 MHz, CDCl_3_): δ = 4.35 (dd, 1H, ^2^*J* = 10.6 Hz, ^3^*J* = 2.5 Hz, –O–C*H*_2_–), 4.44 (dd, 1H, ^2^J = 10.6 Hz, ^3^*J* = 6.5 Hz, –O–C*H*_2_–), 5.26 (dd, 1H, ^3^*J* = 6.5 Hz, 2.5 Hz, –C*H*OH–), 6.81 (d, 1H, ^3^*J* = 8.1, *o*-H to –O–CH_2_–), 6.87 (t, 1H, ^3^*J* = 7.04, *p*-H to –O–CH_2_–), 7.17–7.23 (m, 1H, *p*-H to –CHOH–), 7.34 (d, 1H, ^3^*J* = 7.5 Hz, *o*-H to –CHOH–).

**Benzo[*b*]furan** was purchased from Sigma–Aldrich and used without further purification. For matrix isolation studies, the sample was cooled to −35 °C to reduce its vapor pressure.

### Computational methods

All computations were performed with the Gaussian09 [27] or Cfour suite [28] of programs. All structures were computed employing the M06 density functional [29–30] with doubled (50%) HF-exchange (M06-2X), developed by Truhlar and co-workers, in conjunction with a Dunning-type correlation-consistent double-ζ (zeta) basis set [31] (cc-pVDZ). Single point energies of the DFT structures were evaluated with the coupled cluster method, incorporating singles and doubles as well as perturbative triples and taking into account both valence and core electrons [32–34]. Again, cc-pVDZ was used as the basis set (AE-CCSD(T)/cc-pVDZ). For the elucidation of solvent effects, M06-2X/cc-pVDZ-computations with the Polarization Continuum Model [35] (PCM) were performed, containing radii based on the United Atom Topological Model as implemented in Gaussian09. Tunneling half-lives *τ*_1/2_ of carbenes **5** and **12** were estimated employing a) a simple Eckart barrier methodology [36–37], and b) the one-dimensional Wentzel–Kramers–Brillouin approximation [13–14,37–38].

#### Eckart-barrier approach

For evaluation of the rate constant *k*, the activation barriers of the forward and back reaction, V_f_ and V_b_, were computed, along with the imaginary frequency *ν*_i_ of the transition state and the frequency *ν_ξ_*, corresponding to the reaction coordinate. All energies were vibrationally zero-point corrected, excluding *ν*_i_ and *ν*_ξ_. The transmission probability *P* was computed as


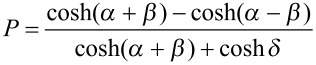
, with the three parameters being


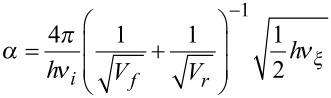
,


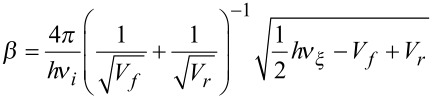
 and


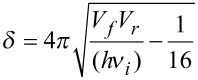
 .

The half-life *τ*_1/2_ was obtained by employing the rate law of first-order kinetics:


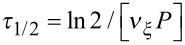
 .

Computations at B3PW91/cc-pVDZ were found to produce reasonable half-lives for PhCOH [21,39–40], for which experimental data are available.

#### Wentzel–Kramers–Brillouin approximation

The intrinsic reaction path, IRP [41], (or minimum energy path, MEP) for each molecule was established at M06-2X/6-311++G(d,p) using the Hessian-based predictor-corrector algorithm [42] as implemented in Gaussian09, with tight convergence criteria. An augmented triple-ζ basis is necessary to achieve an IRP as accurate as possible (as far as the system size permits). The single point energies along the reaction path were vibrationally zero-point corrected by adding the energy contribution of the projected frequencies along the path, i.e., a zero-point correction excluding the frequency *ν*_ξ_ , corresponding to the reaction coordinate. The thus obtained corrected potential along the reaction coordinate ξ was then characterized by an interpolating function *V*(ξ). The attempt frequency of barrier penetration, *ν*_ξ_, was identified by comparing the starting material’s frequencies and the projected frequencies [43] along the IRC. The barrier penetration integral σ between the classical turning points s_1,2_ , where *V*(ξ) = *ε* and


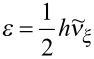
, was then computed as


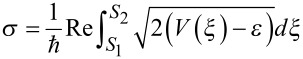
 .

With *σ* at hand, the transition probability *P* could be computed as


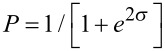
 .

The half-life *τ*_1/2_ was again obtained by employing the rate law of first-order kinetics:


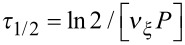
 .

All mathematical operations were carried out with the Mathematica software package [44].

## Supporting Information

File 1 Full matrix isolation spectra.
