# Supplementary material for: Intramolecular hydroxycarbene C–H-insertion: The curious case of (o-methoxyphenyl)hydroxycarbene
Source: Beilstein J Org Chem. 2010 Nov 11;6:1061–9. doi: 10.3762/bjoc.6.121 (PMC3002070; doi:10.3762/bjoc.6.121)
Supplement: File 1 — Full matrix isolation spectra. [file Beilstein_J_Org_Chem-06-1061-s001.pdf]

Supporting Information

for

Intramolecular hydroxycarbene C–H-insertion: The curious case of (*o*-methoxy)phenylhydroxycarbene

Dennis Gerbig, David Ley, Hans Peter Reisenauer and Peter R. Schreiner*

Address: Institut für Organische Chemie, Justus-Liebig University Giessen, Heinrich-Buff-Ring 58, 35392 Giessen, Germany

Email: Dennis Gerbig - Dennis.Gerbig@org.chemie.uni-giessen.de

David Ley - David.Ley@org.chemie.uni-giessen.de

Hans Peter Reisenauer - Hans.P.Reisenauer@org.chemie.uni-giessen.de

Peter R. Schreiner* - prs@org.chemie.uni-giessen.de

Full matrix isolation spectra

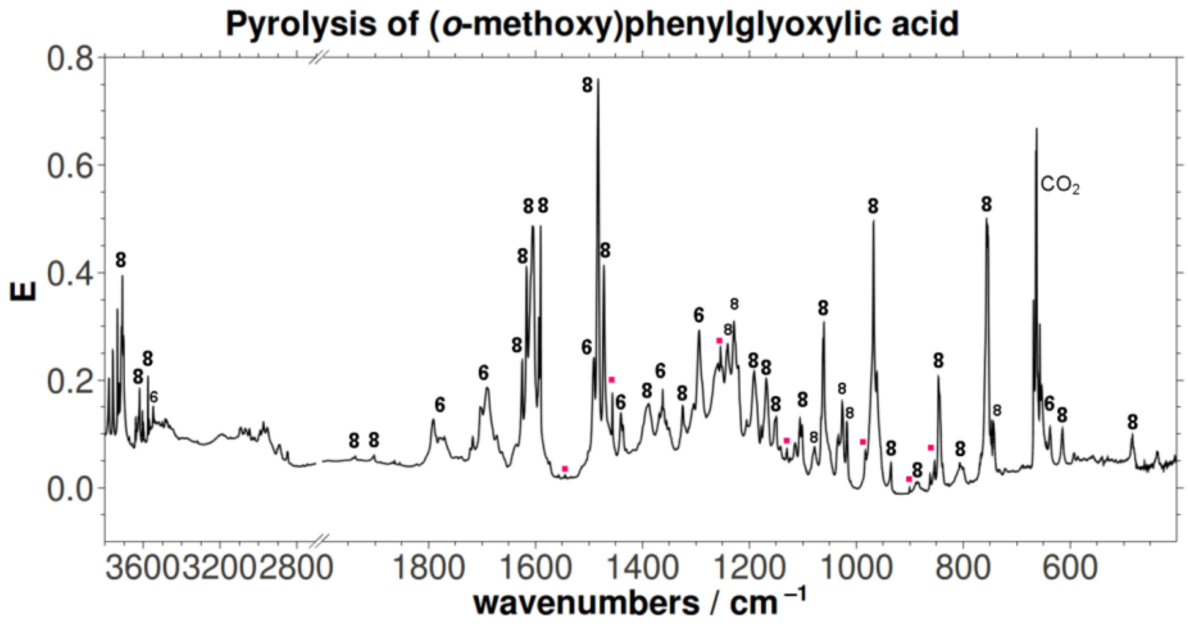

Figure S1: Unmodified matrix IR spectrum (Ar, 11 K) of the pyrolysis (600 °C) of **5**.

Traces of **9** are indicated by magenta dots.

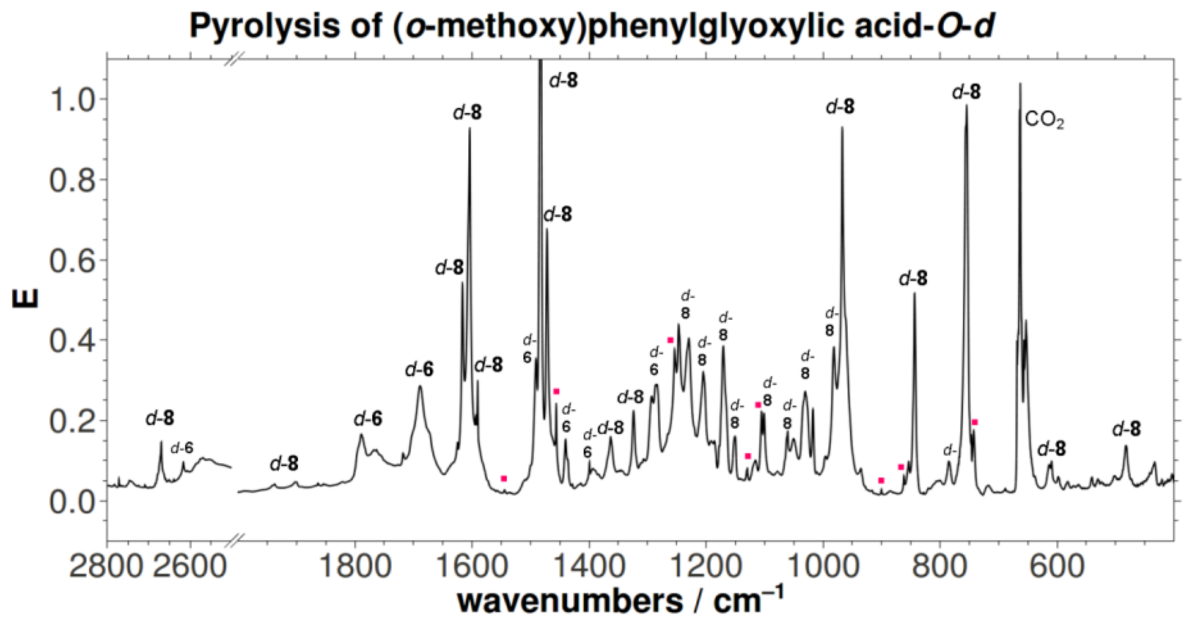

Figure S2: Unmodified matrix IR spectrum (Ar, 11 K) of the pyrolysis (600 °C) of *d*-**5**.

Traces of **9** are indicated by magenta dots.

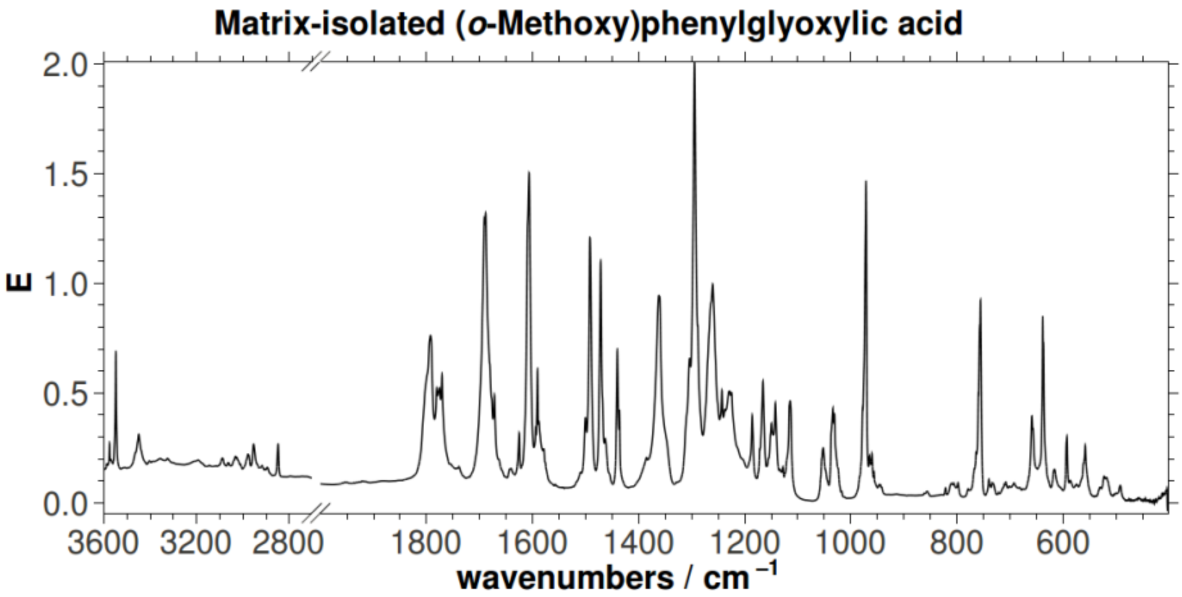

Figure S3: Unmodified matrix IR spectrum (Ar, 11 K) of **6**.

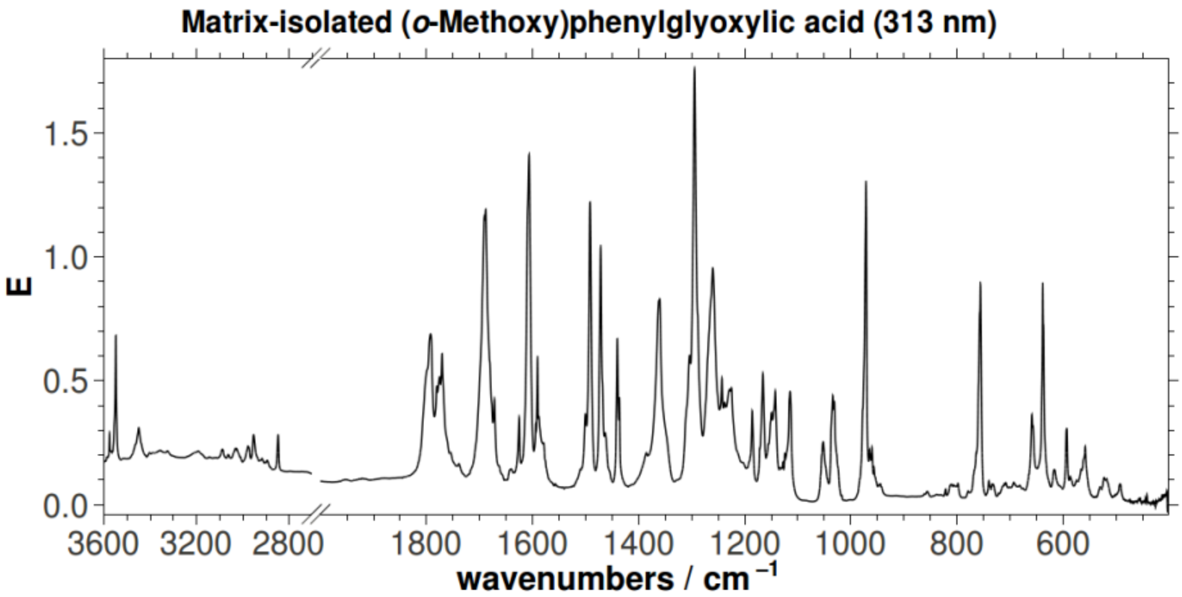

Figure S4: Unmodified matrix IR spectrum (Ar, 11 K) of **6** after irradiation at 313 nm.

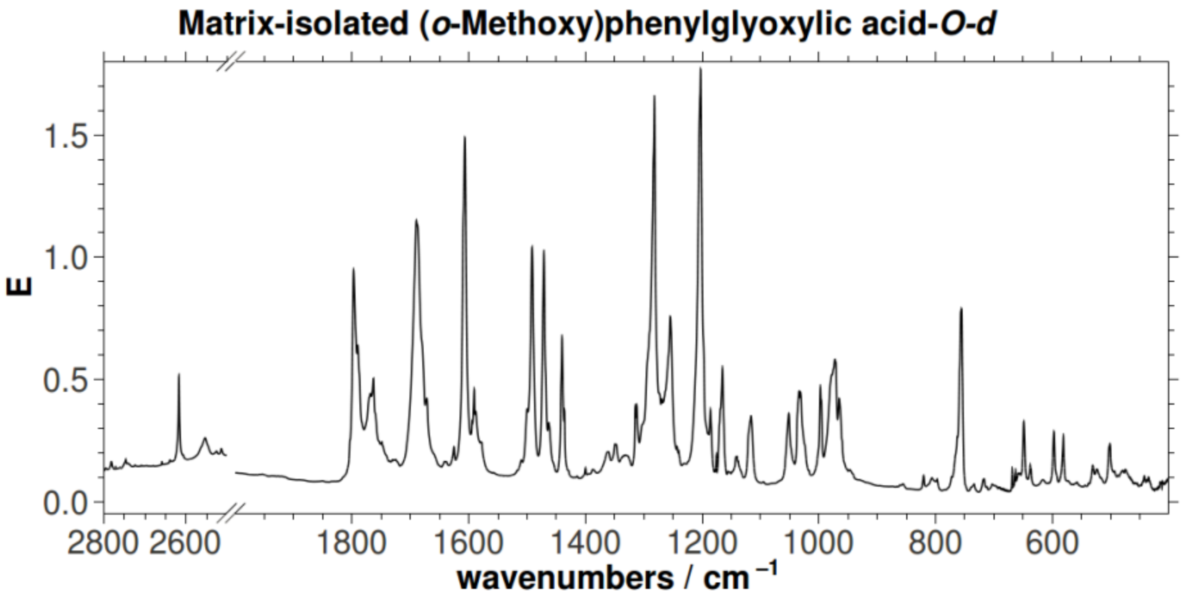

Figure S5: Unmodified matrix IR spectrum (Ar, 11 K) of *d*-**6**.

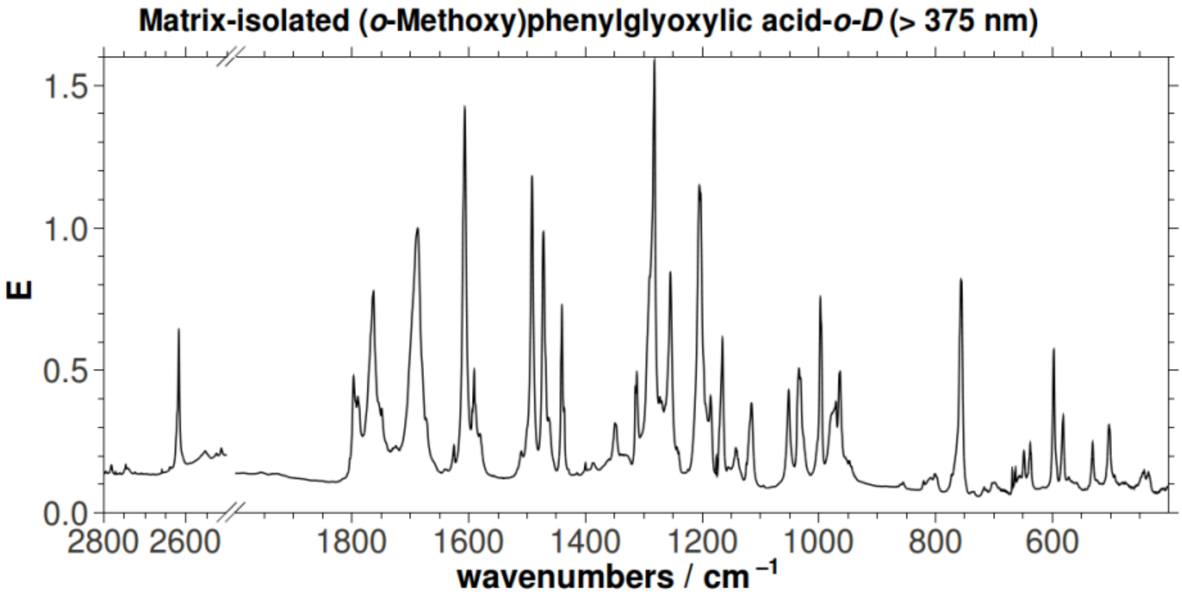

Figure S6: Unmodified matrix IR spectrum (Ar, 11 K) of *d*-**6** after irradiation with wavelengths greater than 375 nm.

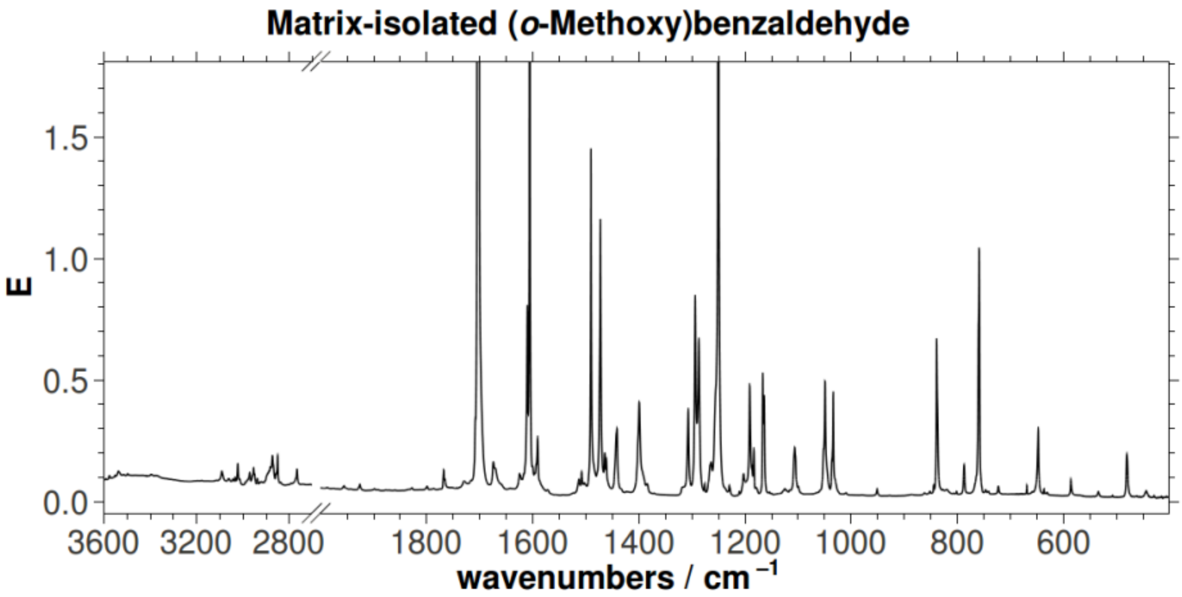

Figure S7: Unmodified matrix IR spectrum (Ar, 11 K) of **7**.

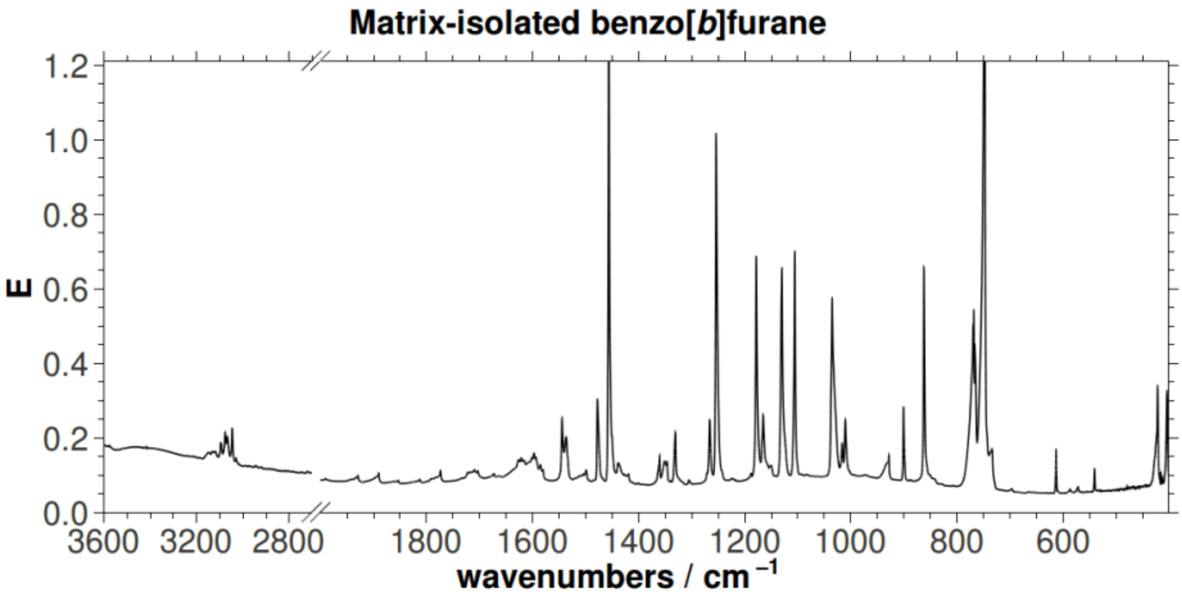

Figure S8: Unmodified matrix IR spectrum (Ar, 11 K) of **9**.

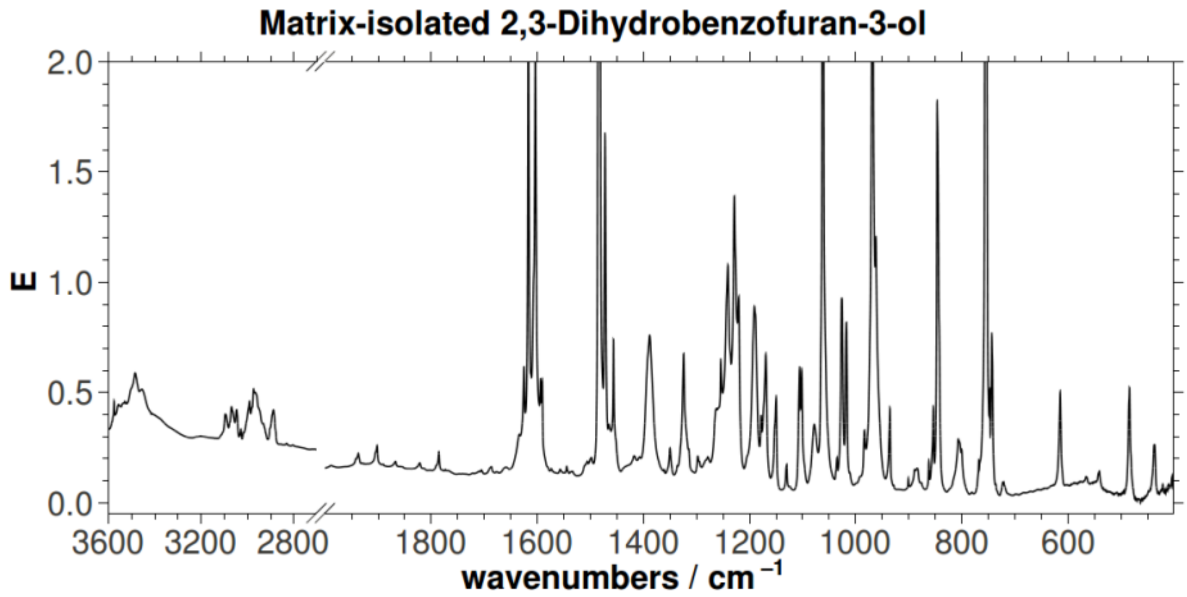

Figure S9: Unmodified matrix IR spectrum (Ar, 11 K) of **8**.

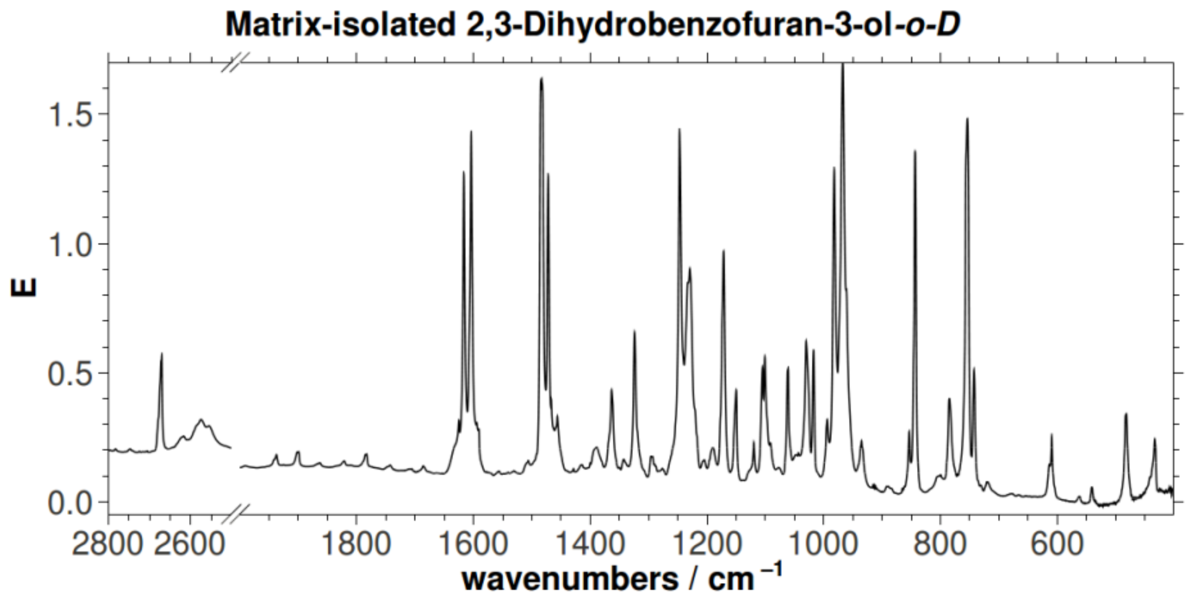

Figure S10: Unmodified matrix IR spectrum (Ar, 11 K) of *d*-**8**.

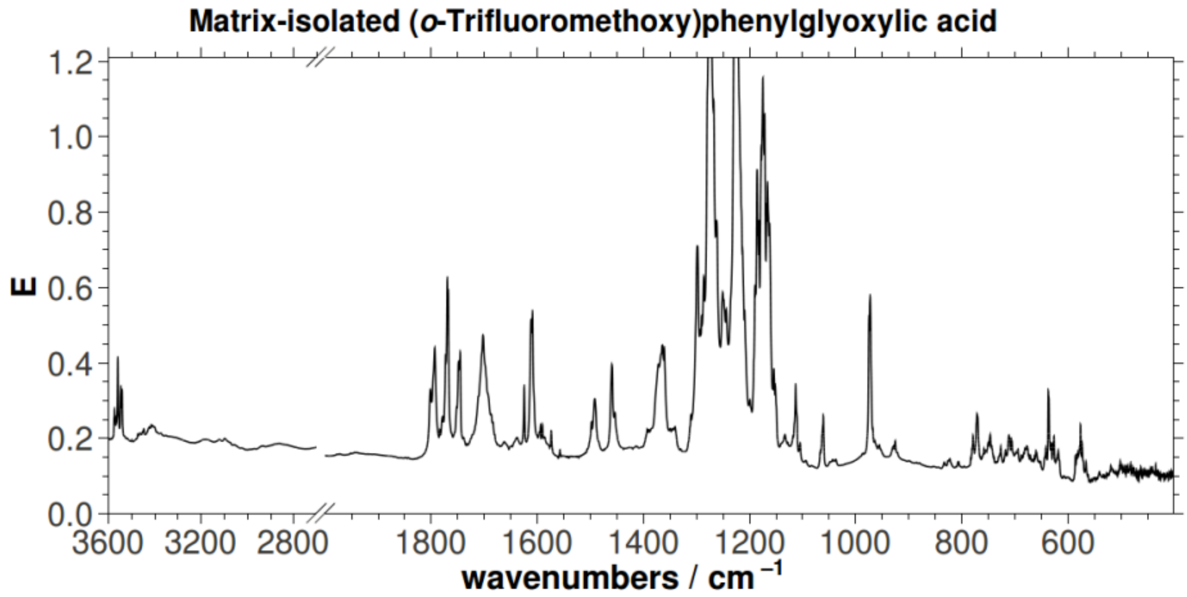

Figure S11: Unmodified matrix IR spectrum (Ar, 11 K) of **10**.

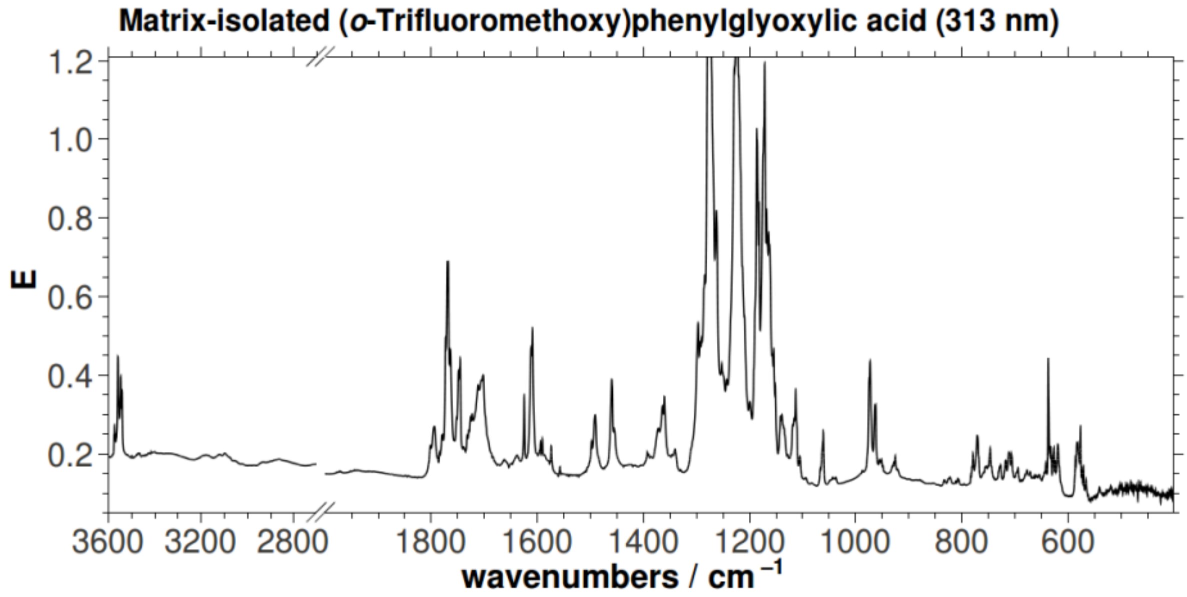

Figure S12: Unmodified matrix IR spectrum (Ar, 11 K) of **10** after irradiation at 313 nm.

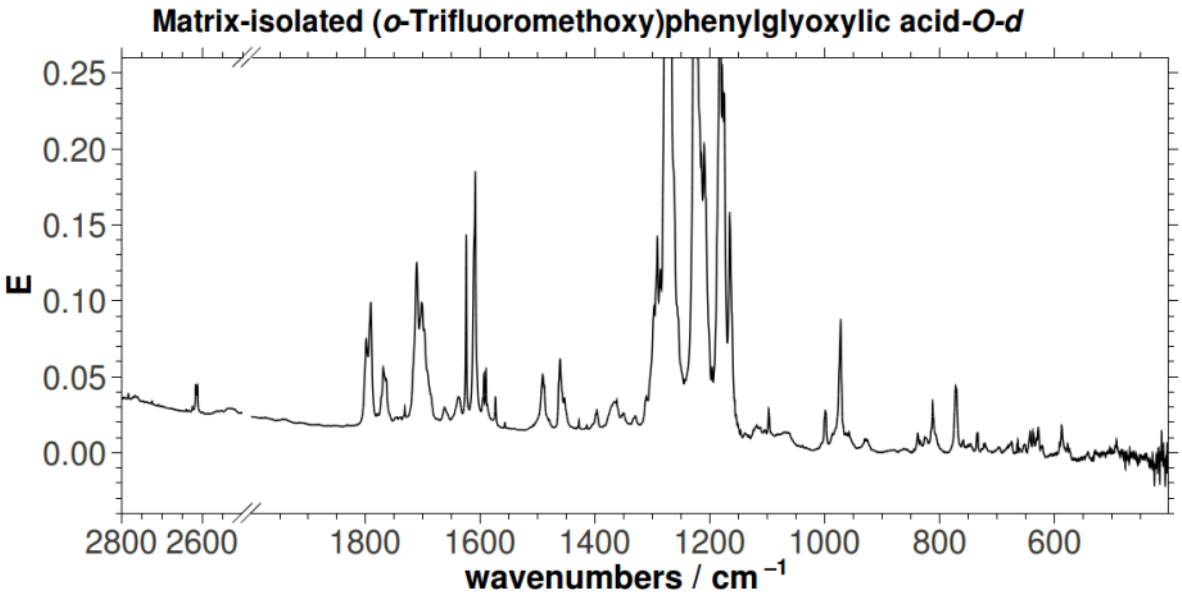

Figure S13: Unmodified matrix IR spectrum (Ar, 11 K) of *d*-**10**.

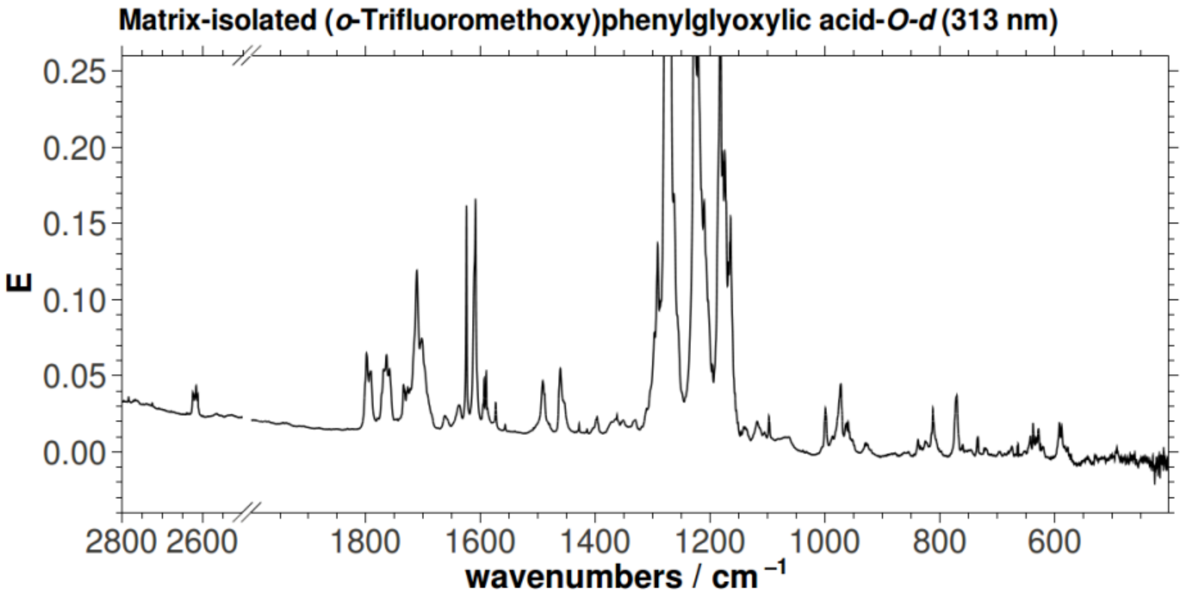

Figure S14: Unmodified matrix IR spectrum (Ar, 11 K) of *d*-**10** after irradiation at 313 nm.

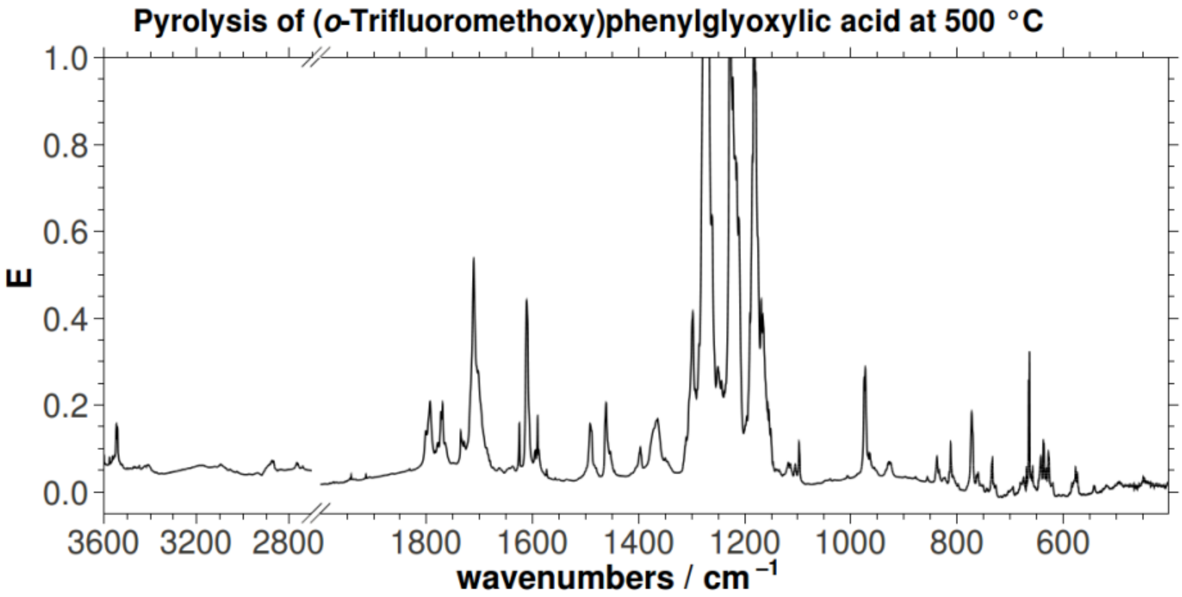

Figure S15: Unmodified matrix IR spectrum (Ar, 11 K) of the pyrolysis (500 °C) of **10**.

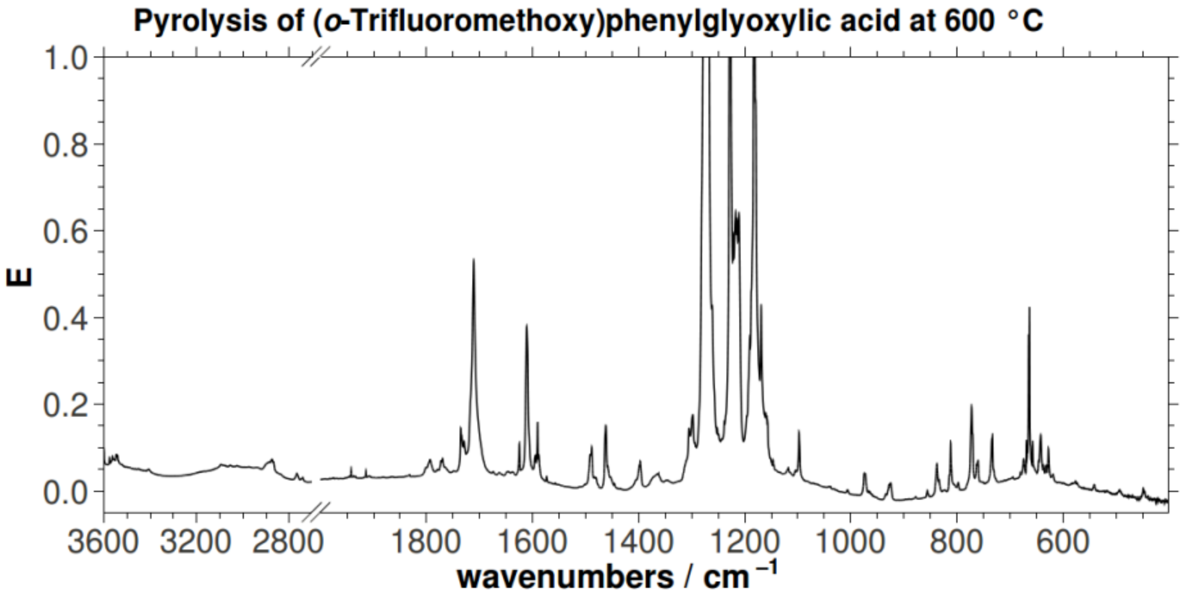

Figure S16: Unmodified matrix IR spectrum (Ar, 11 K) of the pyrolysis (600 °C) of **10**.

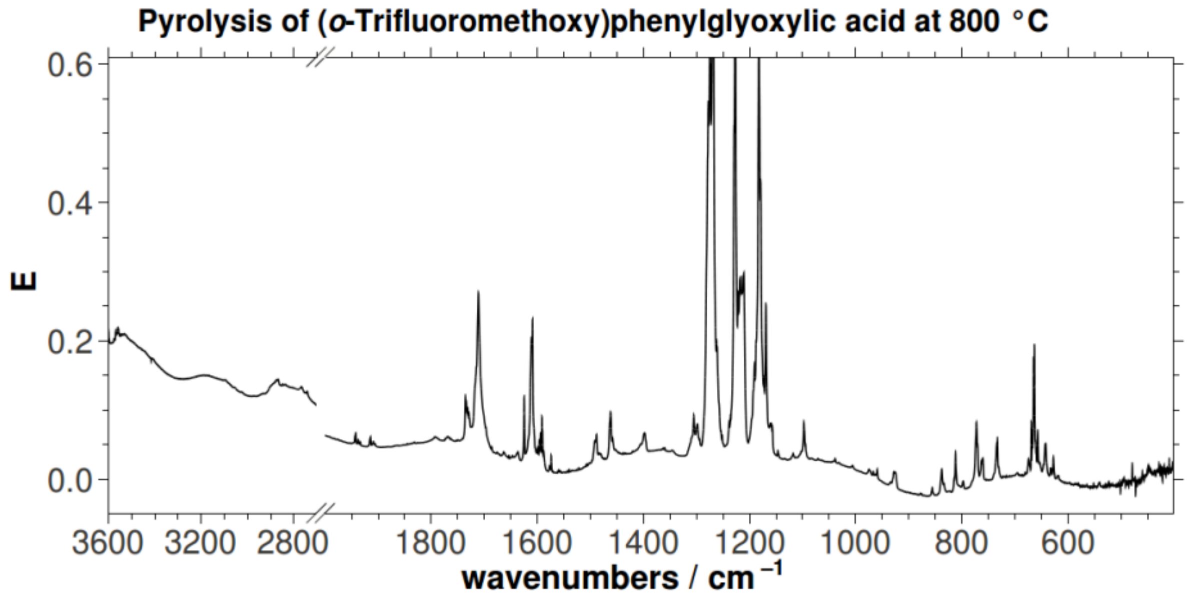

Figure S17: Unmodified matrix IR spectrum (Ar, 11 K) of the pyrolysis (800 °C) of **10**.

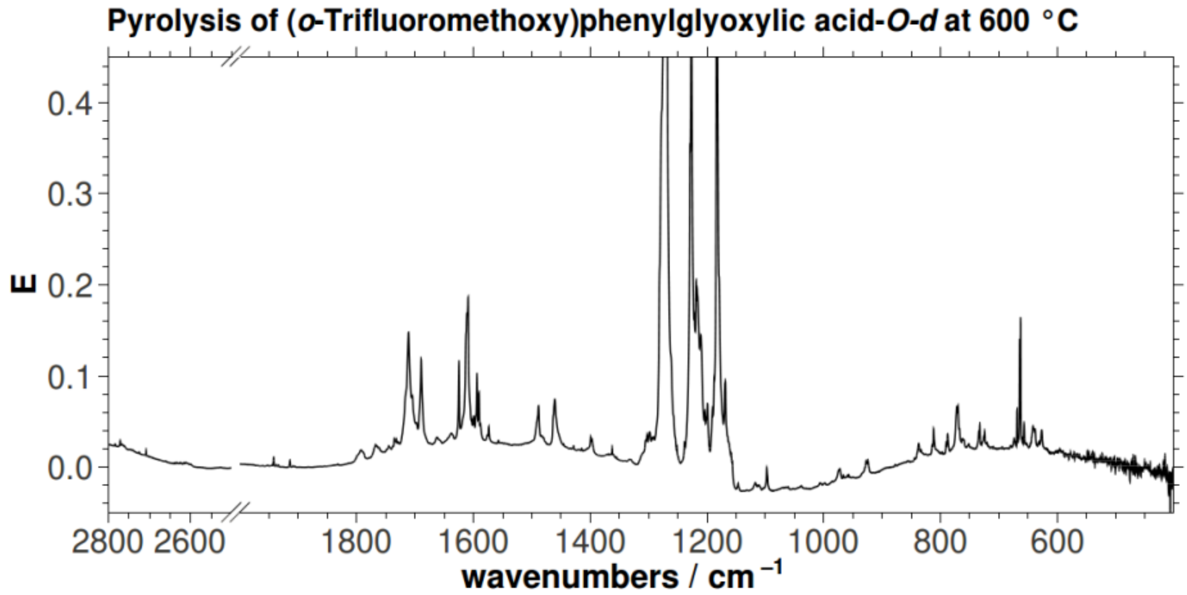

Figure S18: Unmodified matrix IR spectrum (Ar, 11 K) of the pyrolysis (600 °C) of *d*-**10**.

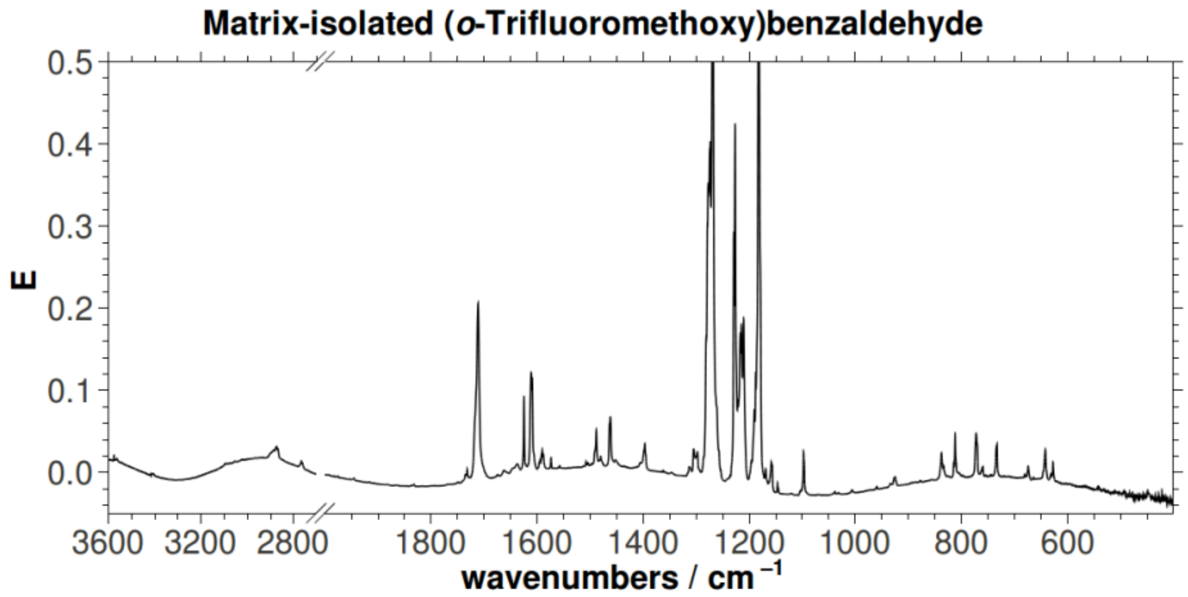

Figure S19: Unmodified matrix IR spectrum (Ar, 11 K) of **11**.
